# Retraction: Dermokine mutations contribute to epithelial-mesenchymal transition and advanced melanoma through ERK/MAPK pathways

**DOI:** 10.1371/journal.pone.0300807

**Published:** 2024-03-26

**Authors:** The PLOS ONE Editors

Following the publication of this article [1], concerns were raised regarding results presented in Fig 2 of [1] that appear similar to results previously published under a CC-BY 4.0 license in [2] by a different author group. Specifically,

- The Fig 2C MUM-8B NC panel in [1] and the Fig 9B Huh-7 SKLB1002/NC mimics panel in [2].
- The Fig 2C MUM-8B shDMKN panel in [1] and the Fig 9B SK-HEP-1 SKLB1002/NC mimics panel in [2].
- The Fig 2D C8161 NC panel in [1] and the Fig 9C SK-HEP-1 Mock panel in [2].
- The Fig 2D C8161 shDMKN panel in [1] and the Fig 9C Huh-7 SKLB1002/miR-101 mimics panel in [2].
- The Fig 2D MUM-8B shDMKN panel in [1] and the Fig 9C SK-HEP-1 SKLB1002/miR-101 mimics panel in [2].

One of the corresponding authors (SI) stated that the panel overlap was the result of a clerical error and provided underlying data for editorial review. However, the data and response provided were not sufficient to resolve the issues raised with this article.

The above concerns call into question the reliability and provenance of the results. Therefore, the *PLOS ONE* Editors retract this article.

MM, SI, and QLW agreed with the retraction and apologize for the issues with the article. WM, ZW, II, MDS, YZ, QW, and JF either did not respond directly or could not be reached.
